# Knowledge of Medical Students and Medical Professionals Regarding Nutritional Deficiencies in Patients with Celiac Disease

**DOI:** 10.3390/nu13061771

**Published:** 2021-05-22

**Authors:** Łukasz Dembiński, Artur Mazur, Mariusz Dąbrowski, Teresa Jackowska, Aleksandra Banaszkiewicz

**Affiliations:** 1 Department of Pediatric Gastroenterology and Nutrition, Medical University of Warsaw, 02-091 Warsaw, Poland; lukasz.dembinski@wum.edu.pl (Ł.D.); aleksandra.banaszkiewicz@wum.edu.pl (A.B.); 2 College of Medical Sciences, University of Rzeszow, 35-310 Rzeszów, Poland; drmazur@poczta.onet.pl; 3 Department of Pediatrics, Center of Postgraduate Medical Education, 01-813 Warsaw, Poland; tjackowska@cmkp.edu.pl

**Keywords:** celiac disease, gluten-free diet, nutritional deficiencies

## Abstract

A gluten-free diet provides relief from symptoms for patients with celiac disease, although there is still a risk of nutritional deficiencies. These patients can potentially consume an excessive amount of fat and insufficient amounts of fiber, iron, vitamin D, and calcium. This study aimed to assess the knowledge of medical students and healthcare professionals in Poland regarding nutritional deficiencies and the prevention of such deficiencies in patients with celiac disease who are on a gluten-free diet. Of the 430 survey participants, 46% did not realize the risk of nutritional deficiencies in patients with celiac disease. The knowledge of the participants was lowest regarding the risk of being overweight or obese. Among the healthcare professionals, an acceptable level of correct answers was provided by only 37% of individuals and was highest for the dietitians’ group. Our results demonstrate the need to improve the education of healthcare professionals concerning nutrition in patients with celiac disease.

## 1. Introduction

Celiac disease (CD) is one of the most common chronic diseases with an increasing prevalence as high as 1–2% [1,2,3,4]. CD is a primary intestinal disease and can result in severe damage to the intestinal mucosa, malabsorption, and consequently, nutritional deficiencies [5]. It is well known that untreated CD may lead to growth failure, iron deficiency and/or vitamin B12 anemia, and osteopenia, etc. [6].

A gluten-free diet (GFD) is the sole treatment for patients with CD. Once diagnosed, patients must adhere to a GFD for life [6]. When a GFD is strictly adhered to, it is clinically extremely effective. In recent years, gluten-free products have been perceived as “very healthy,” and they are commonly consumed by people who do not have CD [7]. However, recent studies have shown that a GFD may also be associated with nutritional deficiencies [8,9]. A systematic review of 35 studies that assessed nutritional deficiencies in children with CD who followed a GFD demonstrated that they were potentially at risk of consuming an excessive amount of fat and insufficient amounts of fiber, iron, vitamin D, and calcium [8]. Alterations in the intake of folate, magnesium, and zinc were also noted. This may contribute to overweight and obesity, increased cardiovascular risk, and to a lower bone density, which is particularly important in the developmental age [10,11].

The knowledge of CD among healthcare workers has been recently studied and was found to be insufficient [12,13,14]. However, an assessment of the knowledge of healthcare professionals (HCPs) concerning nutritional deficiencies in patients with CD who follow a GFD has not been performed. Therefore, this study aimed to complete such an assessment using a questionnaire.

## 2. Materials and Methods

An online survey was designed, using Google Forms platform, to assess the knowledge of medical students and HCPs. The link to it was shared by mail and websites of medical societies. The HCPs group included doctors, dietitians, and nurses. Medical students included senior students of following faculties: medicine, nursing, dietetics, emergency medical services, and physiotherapy. According to their curriculums, they have already been aware of impact of nutrition on CD treatment. The survey contained 12 single or multiple-choice questions that focused on the content of macro- and micronutrients in a GFD and the impact of the diet on the health of patients with CD (Supplementary file). The scope of knowledge and the accuracy of the answers were based on a systematic review of nutritional deficiencies in patients with CD who followed a GFD [8]. The respondents were recruited in Poland, in March and April 2021.

A minimum of 60% (5/8) correct answers for questions regarding nutritional deficiencies was considered an acceptable level of knowledge. In the statistical analyses, the incorrect and inconclusive responses were pooled. Statistical analyses were performed using Statistica version 13.1 software (TIBCO Software Inc., Palo Alto, CA, USA). Multivariate logistic regression model was used to evaluate the associations between acceptable level of knowledge and the following variables: sex, age, experience, providing care for patients with CD, primary medical specialization of doctors, and study field for students. The 95% confidence interval (CI) was used to estimate the precision of the odds ratios (OR) and a *p*-value of <0.05 was considered to be significant.

## 3. Results

Of the 430 survey participants, 66% (283/430) were medical students and 34% (147/430) were HCPs. The detailed characteristics of the study participants are presented in Table 1. Medical students included students of following faculties: medicine, nursing, dietetics, emergency medical services, and physiotherapy.

As many as 46% (196/430) of the respondents did not realize the risk of nutritional deficiencies in patients with CD. An awareness of the risk of nutritional deficiencies was highest among dietitians compared to physicians, nurses, and students: 82% (14/17) (*p* = 0.03; OR 4.02; 95% CI 1.13–14.30) vs. 52% (53/101), 52% (15/29), and 54% (152/283), respectively.

Of all the respondents, 80% (344/430) agreed that a GFD is healthy, although 93% (401/430) recommended that a dietitian should be consulted before introducing the diet. Only 10% (43/430) of respondents rated their knowledge of a GFD as sufficient, the largest proportion of these were dieticians and dietetics students (35% [6/17] and 10% [6/58], respectively).

Regarding patients with CD, 71% (306/430) of respondents recommended vitamin D supplementation, 50% (212/430) recommended micronutrient supplementation, and 16% (67/430) claimed that these patients do not require any supplementation.

The most incorrect answers were provided to questions concerning an increased risk of being overweight or obese, higher saturated fatty acid content, and a higher glycemic index in processed gluten-free products (Table 2). 27% (115/430) of respondents believed that patients with CD should follow a high-calorie GFD.

**Figure 1:**
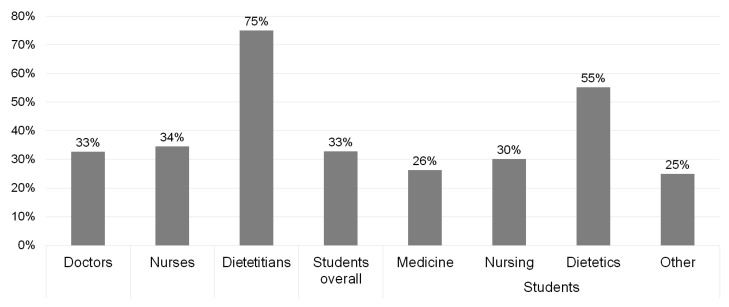
Acceptable level of knowledge (≥60% correct answers) for each occupation.

An acceptable level of correct answers was provided by only 37% (55/147) of HCPs (Table 3). The largest proportion of correct answers was observed in the dietitians’ group (Figure 1). Professional experience of greater than ten years was the only statistically significant risk factor of insufficient knowledge in the doctors’ group (*p* = 0.035). No statistically significant results were obtained regarding the factors influencing the level of knowledge of nurses or dietitians. Among the students surveyed, future dietitians showed a statistically significant higher percentage of correct answers (*p* = 0.024).

## 4. Discussion

The results of this study demonstrate that approximately 46% of participants declared that people with CD who follow a GFD are not at risk of nutritional deficiencies. This corroborates the results of previous studies that assessed the knowledge of HCPs regarding CD. However, none of these studies assessed knowledge strictly regarding nutritional deficiencies associated with a GFD [12,15]. Riznik et al. [12] used a questionnaire that consisted of 22 questions which were divided into three subsections: epidemiology and clinical presentation, diagnostic procedure, and treatment. The authors observed that the knowledge of HCPs (from five European countries: Croatia, Hungary, Germany, Italy, and Slovenia; n = 1381) concerning CD was unsatisfactory. Greater than 50% of the total score was achieved by only 51% of HCPs. Moreover, a study conducted in Romania demonstrated that only one-third of HCPs performed a total IgA test on patients who were diagnosed with CD [15]. The results of the above-mentioned studies suggest that knowledge of HCPs regarding CD is not satisfactory, regardless of the country in which they live.

An unexpected result obtained in the present study was a low level of knowledge regarding nutritional deficiencies among the HCPs who had a longer period of experience in the field—doctors who worked in the field for greater than ten years had a 70% lower chance of having an acceptable level of knowledge than those who worked in the field for less than ten years. This observation is particularly worrying given the fact that three-quarters of the HCPs surveyed cared for patients with CD. The results of previous studies are dichotomous. Several authors observed that young physicians had improved knowledge of CD compared with older physicians [12,13], however, the authors of other studies did not observe this [16]. Previous studies on the impact of time regarding the medical knowledge of staff (not of CD) demonstrated mixed results. The results of several studies suggested a rapid loss of knowledge, particularly theoretical knowledge [17,18,19]. Conversely, the results of a separate study showed that the importance of experience gained over time should not be underestimated [20]. The results of the present study indicate the need to intensify the continuous education of HCPs to improve care for patients with CD.

The results of our study demonstrate a significant disproportion concerning the level of knowledge of current and future doctors compared with that of dietitians. This may be due to other educational priorities, as demonstrated in a separate study [21]. However, as nutrition is an essential part of the therapy for many diseases, it should not be neglected in the medical education process [22].

Most of the respondents believed that a GFD is not associated with being overweight or obese. In patients with CD, a GFD heals the intestinal mucosa, decreases intestinal permeability, and improves a patient’s condition, including their appetite [23]. If malnutrition is present when CD is diagnosed, a GFD can improve the nutritional status of the patient. In such cases, weight gain is beneficial and can aid in catching up on growth. However, when catch-up growth is achieved, a GFD may lead to a patient becoming overweight or obese. This can be the case for all diets, particularly when they contain additional saturated fatty acids and simple carbohydrates, as most gluten-free processed foods do.

We believe that this result is associated with a separate observation in the present study: greater than 80% of the participants perceived the GFD as healthy. Many individuals believe that, in general, a GFD is a “healthy diet”, thus, it cannot be associated with being overweight or obese [24]. On the contrary, “healthy” generally means that weight gain is avoided [25]. In our opinion, this is the most probable explanation for the results we obtained during the present study.

In this study, only 10% of participants believed that their knowledge regarding a GFD was sufficient. This was three times lower than the actual number of HCPs who had sufficient knowledge. In similar studies conducted on nutritional knowledge, approximately half of respondents believed their knowledge was sufficient [26,27]. Our results indicate a lack of confidence in GFD counseling competence among HCPs. This may favor a negative trend of HCPs who provide GFD counseling. Our results demonstrate that there is a requirement to provide HCPs with knowledge concerning nutritional deficiencies in patients with CD who follow a GFD. To our knowledge, this is the first study to assess the knowledge of medical students and HCPs regarding nutritional deficiencies in patients with CD who follow a GFD. The relatively low number of respondents, particularly among nurses and dietitians, may be considered as a limitation of this study. Only Polish students and HCPs participated in the survey and it is questionable to directly extrapolate this data to the global population, however the curricula are similar and GFD is commonly used in the Western countries. There is also a certain overrepresentation of women and pediatricians among the respondents. This may result from the subject of the study itself, CD is most often diagnosed in developmental age, i.e., mainly by pediatricians. On the other hand, the pediatric specialization is statistically more often chosen by women worldwide [28]. Taking these two facts together women pediatricians are probably the most interested in CD and GFD group among doctors.

## 5. Conclusions

Healthcare professionals and medical students have insufficient knowledge of the risk of nutritional deficiencies in patients with CD. Medical education should include the diagnostic methods of CD as well as counseling on the appropriate composition of a GFD. Also, longer working doctors should pay more attention to continuous education to improve care for patients with CD.

## Figures and Tables

**Table 1:** Basic characteristics of the study participants.

Parameter	Variable	Result, n (%)
Sex	Male	67 (15.58%)
	Female	363 (84.42%)
Age	≤30 years	317 (73.72%)
	>30 years	113 (26.28%)
Occupation	Medical students Medical professionals	283 (65.81%) 147 (34.19%)
	**Medical Students (n = 283)**	
Studies	medicine nursing dietetics other medical faculties	152 (53.71%) 53 (18.73%) 58 (20.49%) 20 (7.07%)
**Medical professionals (n = 147)**		
Occupation	doctors nurses dietitians	101 (68.71%) 29 (19.73%) 17 (11.56%)
Workplace	hospital outpatient healthcare	115 (78.23%) 32 (21.77%)
Experience	≤10 years >10 years	88 (59.86%) 59 (40.14%)
Provides care for patients with celiac disease	yes no	112 (76.19%) 35 (23.81%)
Primary medical specialization of doctors	pediatric other	87/101 (86.14%) 14/101 (13.86%)

**Table 2:** Distribution of correct answers.

No.	Statements	Total, n = 430 % (n)	Medical Professionals, n = 147 % (n)	Medical Students, n = 283 % (n)
Q1.	Patients with celiac disease should follow a gluten-free diet that contains more calories compared to healthy people.	73.26% (315/430)	80.95% (119/147)	69.26% (196/283)
Q2.	Patients with celiac disease can be exposed to nutritional deficiencies by following a gluten-free diet.	54.42% (234/430)	55.78% (82/147)	53.71% (152/283)
Q3.	Individuals with celiac disease who follow a gluten-free diet can become overweight or obese.	25.12% (108/430)	31.97% (47/147)	21.55% (61/283)
Q4.	A gluten-free diet favors eating fewer complex carbohydrates.	60.70% (261/430)	53.74% (79/147)	64.31% (182/283)
Q5.	Gluten-free processed foods contain more saturated fat than their gluten-containing counterparts.	26.98% (116/430)	26.53% (39/147)	27.21% (77/283)
Q6.	Gluten-free processed foods contain more dietary fiber than their gluten-containing counterparts.	47.67% (205/430)	45.58% (67/147)	48.76% (138/283)
Q7.	The glycemic index of gluten-free processed foods is higher compared with their gluten-containing counterparts.	37.91% (163/430)	42.18% (62/147)	35.69% (101/283)
Q8.	All patients with celiac disease should have regular assessment of vitamin D levels, regardless of their supplementation.	60.23% (259/430)	66.67% (98/147)	56.89% (161/283)

**Table 3:** Level of knowledge among study participants and odds ratios (OR) with 95% confidence interval (CI) (in parentheses) of having acceptable level of knowledge (≥60% correct answers). Significant differences marked in *italic*.

Parameter	Variable	Acceptable Level of Knowledge	OR (95% CI)	*p* Value
**Doctors, *n* = 110**				
Sex	Male	19.05% (4/21)	reference	-
	Female	36.25% (29/80)	1.44 (0.40–5.15)	0.576
Age	≤30 years	25.00% (4/16)	reference	-
	>30 years	34.12% (29/85)	2.43 (0.65–9.03)	0.185
Experience	≤10 years >10 years	41.67% (25/60) *19.51% (8/41)*	reference *0.33 (0.12–0.92)*	- *0.035*
Provides care for patients with celiac disease	No Yes	14.29% (2/14) 35.63% (31/87)	reference 2.01 (0.37–10.94)	- 0.419
Primary medical specialization of doctors	Other Pediatric	14.29% (2/14) 35.63% (31/87)	reference 1.85 (0.34–10.23)	- 0.480
**Students, *n* = 283**				
Sex	Male	33.33% (15/45)	reference	-
	Female	32.77% (78/238)	0.70 (0.34–1.45)	0.335
Studies	Other medical faculties	25.00% (5/20)	reference	-
	Medicine	26.32% (40/152)	0.99 (0.33–2.94)	0.983
	Nursing	30.19% (16/53)	1.30 (0.40–4.20)	0.657
	Dietetics	*55.17% (32/58)*	*3.72 (1.19–11.60)*	*0.024*

## Data Availability

Original data are available from the first author upon request.

## Supplementary Materials

The following are available online at https://www.mdpi.com/article/10.3390/nu13061771/s1, Table S1: A survey on a gluten-free diet in patients with celiac disease – part evaluating the knowledge of the respondents.
