# Supplementary material for: Knowledge of Medical Students and Medical Professionals Regarding Nutritional Deficiencies in Patients with Celiac Disease
Source: Nutrients. 2021 May 22;13(6):1771. doi: 10.3390/nu13061771 (PMC8224609; doi:10.3390/nu13061771)
Supplement: Supplementary file 1 [file nutrients-13-01771-s001.zip › nutrients-1222234-supplementary.pdf]

Table S1. A survey on a gluten-free diet in patients with celiac disease – part evaluating the knowledge of the respondents

In your opinion, is a gluten-free diet healthy?	
My knowledge of a gluten-free diet is sufficient	
Patients with celiac disease should follow a gluten-free diet that contains more calories compared to healthy people.	
Patients with celiac disease can be exposed to nutritional deficiencies by following a gluten-free diet.	
Individuals with celiac disease who follow a gluten-free diet can become overweight or obese.	
A gluten-free diet favors eating fewer complex carbohydrates.	
Gluten-free processed foods contain more saturated fat than their gluten-containing counterparts.	
Gluten-free processed foods contain more dietary fiber than their gluten-containing counterparts.	
The glycemic index of gluten-free processed foods is higher compared with their gluten-containing counterparts.	
All patients with celiac disease should have regular assessment of vitamin D levels, regardless of their supplementation.	
The use of a gluten-free diet in a patient with celiac disease does not require a dietician's consultation due to the widespread access to gluten-free products.	
Patients with celiac disease should be advised:	a) supplementation with multivitamin drugs<br>b) vitamin D supplementation<br>c) consuming products enriched with micronutrients<br>d) a gluten-free diet alone is sufficient
Deficiencies of which micronutrients may occur in patients with celiac disease following a gluten-free diet?	a) iron<br>b) calcium<br>c) zinc<br>d) magnesium<br>e) copper<br>f) iodine
Deficiencies of which vitamins can occur in patients with celiac disease following a gluten-free diet?	a) vitamin A<br>b) vitamin E<br>c) vitamin C<br>d) vitamin D<br>e) vitamin B1<br>f) vitamin B12<br>g) folic acid
